# Exploring the Lamina Terminalis: A Stepwise Anatomical Comparison of Pterional and Orbitozygomatic Craniotomy Approaches

**DOI:** 10.3390/life15121804

**Published:** 2025-11-25

**Authors:** Merih C. Yilmaz, Yunus E. Durmus

**Affiliations:** 1 Department of Neurosurgery, VM Medical Park Hospital, 55200 Samsun, Turkey; 2 Department of Neurosurgery, Ondokuz Mayis University Faculty of Medicine, 55270 Samsun, Turkey; dr.emredurmus@gmail.com

**Keywords:** lamina terminalis, neuroanatomy, pterional approach, orbital craniotomy

## Abstract

**Background/Objectives:** The lamina terminalis is an important anatomical structure located at the anterior wall of the third ventricle and surrounded by critical neurovascular elements. Precise and safe surgical access to this region requires detailed anatomical knowledge and mastery of skull base approaches. This study aims to anatomically illustrate and compare the pterional, one-piece orbitozygomatic, and two-piece orbitozygomatic craniotomies as approaches to the lamina terminalis cistern. The objective is to provide a comprehensive photographic guide to support neurosurgeons and residents in learning these techniques. **Methods:** Two formalin-fixed, silicone-injected adult cadaveric heads were dissected under an operating microscope. Standard pterional, one-piece orbitozygomatic, and two-piece orbitozygomatic approaches were performed. Key anatomical landmarks and surgical steps were documented photographically. Osteotomies, dural openings, and basal drilling techniques were systematically applied to maximize exposure of the lamina terminalis region while preserving critical neurovascular structures. **Results:** The results demonstrate that all three approaches effectively expose the lamina terminalis cistern and related vascular structures. By highlighting the trajectory, osteotomies, and neurovascular anatomy, the anatomical dissections provide valuable visual guidance. These findings are intended to support neurosurgical education by illustrating the strengths of each approach in a comparative context. **Conclusions:** This study offers detailed anatomical visualization of three key approaches to the lamina terminalis. The stepwise dissections and high-resolution images are intended as an educational guide to assist neurosurgeons and residents in mastering these techniques. Such anatomical understanding is vital for safe, effective surgical interventions involving the anterior skull base and vascular pathologies.

## 1. Introduction

The lamina terminalis is a significant component of brain anatomy, positioned at the front of the third ventricle. It functions as a slender, vertical membrane stretching from the rostral end of the anterior commissure to the optic chiasm. As a fundamental element of the diencephalon, it plays a crucial role in the arrangement of the ventricular system and adjacent brain structures.

Anatomically, the lamina terminalis constitutes the front boundary of the third ventricle and is situated directly behind the frontal lobes. It is laterally bordered by the anterior columns of the fornix and extends continuously into the hypothalamus.

Besides its physiological functions, the lamina terminalis serves as a key landmark for neurosurgeons due to its closeness to significant brain structures. For effective surgical planning, a thorough understanding of its anatomy is crucial, especially when employing pterional and orbitozygomatic approaches.

Pterional craniotomy, also known as fronto-temporo-sphenoidal craniotomy, remains the primary approach in contemporary neurosurgical practice for addressing cranial pathologies [1]. In 1975, Yaşargil introduced a pioneering modification to the conventional technique, emphasizing limited craniotomy scope, removal of two-thirds of the sphenoid wing, and opening the sylvian fissure and anterior cisterns to minimize frontal lobe retraction [2].

The orbitozygomatic approach serves as an extension of pterional craniotomy, functioning as an additional technique for addressing anterior skull base and orbital lesions. This approach offers surgeons a valuable means of accessing diverse vascular and oncological pathologies without introducing additional morbidity. Hakuba’s orbitozygomatic infratemporal approach in 1986, followed by Al-Mefty’s cranio-orbitozygomatic approach [3,4], marked significant milestones. Zabramski’s introduction of the two-piece cranio-orbitozygomatic approach in 1998 brought an added advantage in widening the surgical field for anterior skull base procedures [5]. Furthermore, the advancement of minipterional and mini-orbitozygomatic techniques has lessened the invasiveness of craniotomy procedures, offering practical alternatives for neurosurgeons engaged in neurovascular and neuro-oncological surgeries [6].

This study systematically visualized pterional, one-piece, and two-piece cranio-orbitozygomatic approaches. By adhering to surgical principles and photographically documenting the modifications of each technique, the lamina terminalis cisterna was reached. This microsurgical neuroanatomical study is intended to contribute to neurosurgeons and residents as a photographic guide.

## 2. Materials and Methods

This anatomical study was conducted at the Microsurgery-Neuroanatomy Laboratory, Department of Neurosurgery, between April and October 2021. Ethical approval was obtained from the Ethics Committee. Permission for the use and publication of cadaveric images was obtained through institutional guidelines. Following the injection of colored silicone, two postmortem human head specimens were immersed in a 10% formalin solution for a minimum of one year to ensure adequate preservation. During intervals between dissections, the cadavers were stored in a 70% alcohol solution at room temperature.

Dissections were performed utilizing a surgical microscope at magnifications of 4× and 40×. The tools employed included dura stop, bone drill and insert, and a microsurgery set consisting of micro forceps, micro hook, micro scissors, scalpel, micro and macro dissector, various sizes of metal spatula, and aspirator. Throughout the dissections of surgical approaches, each stage was meticulously documented using a camera equipped with an 18–55 mm telephoto lens and a 100 mm macro lens.

The specimens were stored in optimal conditions to maintain structural integrity, ensuring the reliability of the findings. The rigorous methodology employed in specimen preparation and the use of advanced microsurgical instruments contribute to the robustness of this neuroanatomical study. Comprehensive photographic documentation provides a visual record of each stage of the dissections, enhancing the reproducibility and scientific value of the study.

## 3. Results

### 3.1. Pterional Craniotomy

#### 3.1.1. Incision

**Figure 1:**
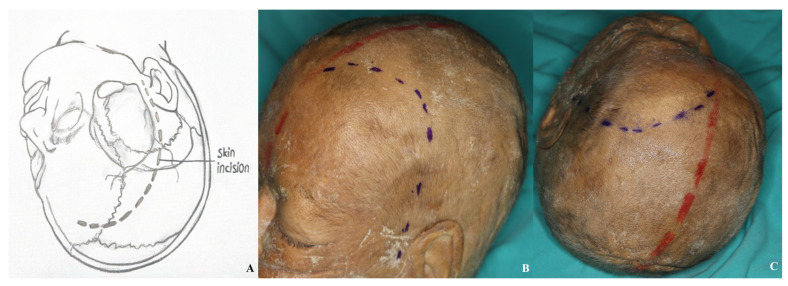
Incision line for pterional craniotomy with all anatomical landmarks visible [(**A**) demonstration, (**B**,**C**) incision view; red line: midline; blue line: incision].

The incision should commence at the superior margin of the zygomatic arch, just anterior to the tragus, and should extend in an anterior arc toward the midline, taking care not to cross the hairline. In certain conditions, such as temporal lobe pathologies or middle cerebral artery aneurysms, the medial extent of the incision may not need to reach the midline. To minimize the risk of injury to the facial nerve and the superficial temporal artery, the lateral limit of the incision should remain within 1 cm of the tragus [Figure 1].

**Figure 2:**
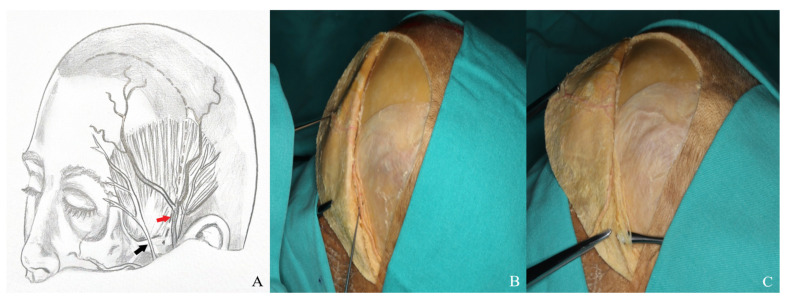
(**A**) Course of the superficial temporal artery [red arrow] and facial nerve [black arrow]. (**B**) The superficial temporal artery was visualized on the skin flap and elevated using a hook. (**C**) The facial nerve and its branches were dissected and mobilized with a dissector.

It is important to exercise caution during dissection to avoid injury to the superficial temporal artery and the facial nerve [Figure 2].

#### 3.1.2. Interfacial, Subfascial Dissection Technique and Temporal Muscle Elevation

**Figure 3:**
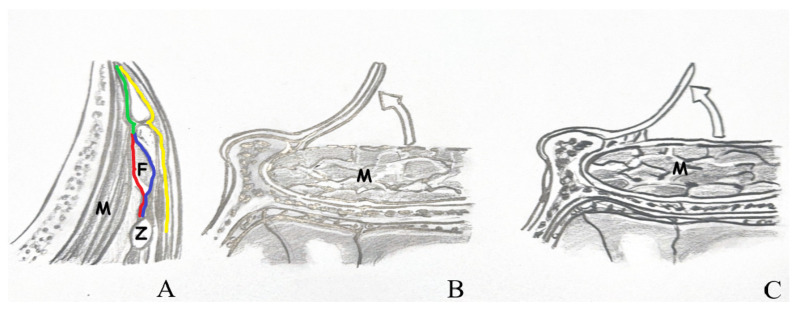
(**A**) View of the temporal muscle fascia. (**B**) Subfascial dissection. (**C**) Interfacial dissection [Z: zygomatic arch; F: fat pad; M: temporal muscle; red line: deep layer of deep temporal fascia; blue line: superficial layer of deep temporal fascia; green line: deep temporal fascia; yellow line: superficial temporal fascia].

The temporalis muscle is enveloped by the superficial temporal fascia, which consists of two distinct layers: superficial and deep. Situated between these layers is an interfacial fat pad, through which branches of the facial nerve traverse. In the interfacial surgical approach, dissection of the superficial temporal fascia should commence in a vertical direction, parallel to the superior temporal line, approximately 1.5 to 2 cm posterior to the orbital rim. Upon elevation of the superficial fascia overlying the temporalis muscle, the interfacial fat pad and the underlying deep temporal fascia become visible. These fat pads, along with the superficial fascia, should then be carefully elevated. Conversely, the subfascial technique involves lifting both the superficial and deep temporal fascia layers en bloc from the temporalis muscle, extending from the superior temporal line to the frontal pericranium [Figure 3].

**Figure 4:**
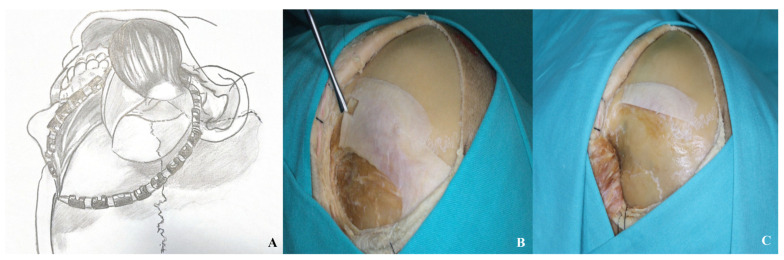
After initial dissection from the keyhole with a dissector, the temporalis muscle was elevated in a subperiosteal plane and retracted inferiorly [(**A**) demonstration, (**B**,**C**) cadaver appearance].

The removal of the temporalis muscle during the pterional approach is performed in two distinct stages. In the initial stage, an incision is made parallel to the superior temporal line, approximately 1.5 to 2 cm inferior to it, extending from anterior to posterior. This technique preserves a superior muscle flap to facilitate later reattachment of the temporalis muscle. In the second stage, the muscle is elevated by subperiosteal dissection from the underlying bone using an elevator. In the orbitozygomatic approach, dissection of the temporalis muscle begins at the inferior margin of the incision and proceeds anteriorly toward the inferior temporal line. A muscle flap is similarly preserved at the superior temporal line to allow for re-suturing. The dissection is continued in a subperiosteal plane inferiorly, following the contour of the zygomatic arch [Figure 4].

#### 3.1.3. Craniotomy

The goal of a pterional craniotomy is to expose the anterior Sylvian fissure. This requires four burr holes. The first, known as the keyhole (McCarty), is placed anterior to the superior temporal line and above the fronto-zygomatic suture. It is a key landmark for accessing the anterior and middle cranial fossae with minimal brain retraction.

**Figure 5:**
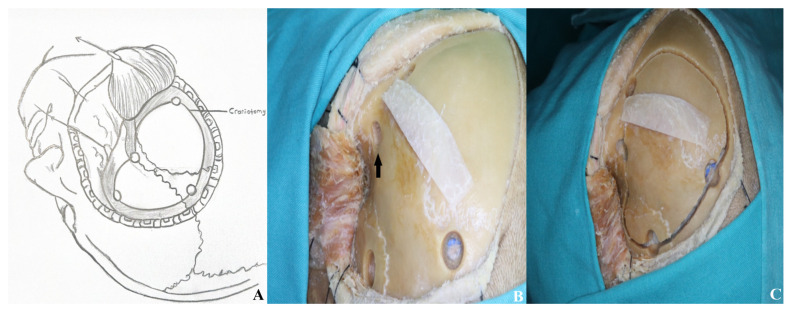
The initial burr hole was made at the McCarty point [black arrow], known as the “keyhole,” followed by three additional burr holes. A pterional craniotomy was then completed to elevate the bone flap [(**A**) schematic, (**B**,**C**) cadaver visualization].

The second hole is made in the frontal bone near the superior orbital rim, avoiding the frontal sinus, and should be 3–4 cm from the first. The third is placed below the superior temporal line, near or just anterior to the coronal suture. The fourth is located on the squamous temporal bone, posterior to the spheno-temporal suture. Craniotomy is completed by connecting the burr holes with osteotomies [Figure 5].

#### 3.1.4. Basal Drilling

For optimal exposure of the anterior and middle cranial base with minimal cerebral retraction, resection of the orbital roof, residual squamous portion of the temporal bone, and the medial segment of the sphenoid wing is necessary. This facilitates access to the carotid cistern and enhances brain relaxation by enabling cerebrospinal fluid drainage following cisternal opening.

**Figure 6:**
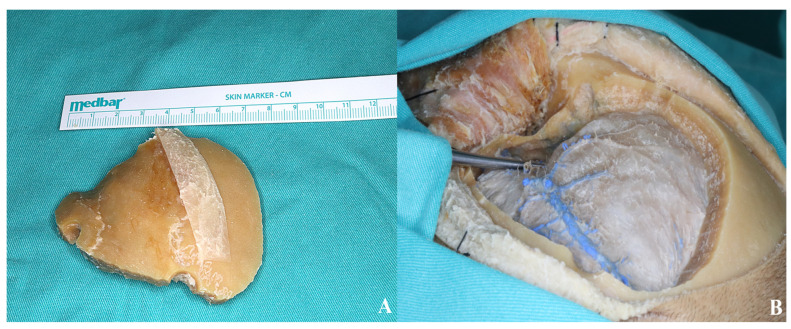
(**A**) Bone flap. (**B**) The thinned sphenoid ridge is demonstrated following basal drilling with the aid of a dissector.

During sphenoid wing resection, the orbital meningeal artery is typically encountered at the superolateral aspect of the superior orbital fissure. This vessel often bleeds and should be coagulated to complete the resection safely [Figure 6].

#### 3.1.5. Dura Incision

**Figure 7:**
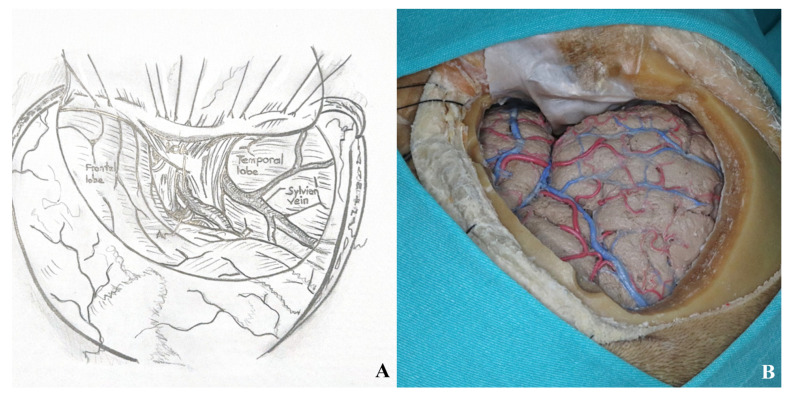
After the dural incision was made, the dura was elevated and suspended. (**A**). Illustration, (**B**). Cadaver footage.

The dural incision is made in a C-shaped fashion, extending from the second burr hole to the temporal base, curving anteriorly toward the zygoma [Figure 7].

Sylvian fissure dissection begins at the frontal operculum, typically at the pars triangularis, where the fissure is widest. Opening the carotid cistern allows cerebrospinal fluid drainage, reducing intracranial pressure and facilitating brain relaxation. This also enables proximal control by exposing the internal carotid artery.

Superficial Sylvian veins must be preserved, especially those draining into the temporal lobe. Frontal draining veins may be sacrificed if necessary.

**Figure 8:**
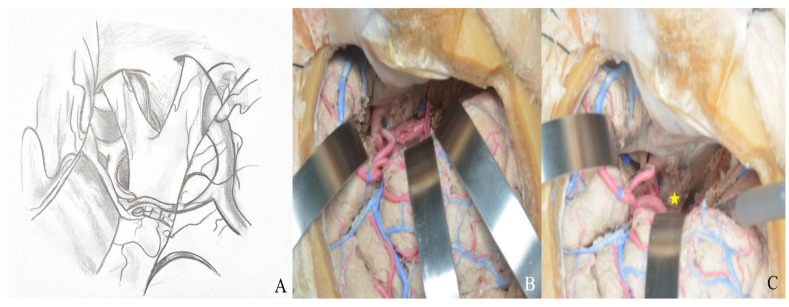
(**A**) Exposure of the lamina terminalis. (**B**) After Sylvian fissure dissection, retractors provided access to the anterior and middle skull base. (**C**) The lamina terminalis cistern was reached by removing the anterior communicating artery using a dissector [yellow star].

Dissection between the frontal and temporal lobes exposes the middle cerebral artery and its branches. After opening the carotid cistern, dissection proceeds medially toward the chiasmatic and lamina terminalis cisterns [Figure 8].

### 3.2. Orbitozygomatic Approach

#### 3.2.1. Incision

**Figure 9:**
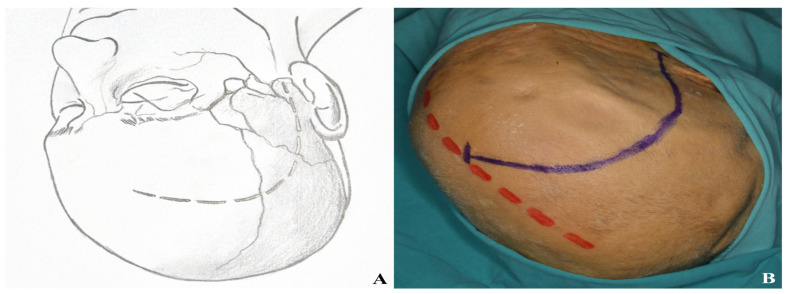
Incision plan for the one-piece orbitozygomatic approach [(**A**) incision sketch, (**B**) cadaver view: red line indicating midline; blue line indicating incision].

The skin incision resembles that of a pterional craniotomy but curves slightly posteriorly toward the midline. It begins 1 cm anterior to the tragus at the level of the zygomatic arch and extends toward the contralateral orbit, remaining behind the hairline [Figure 9].

**Figure 10:**
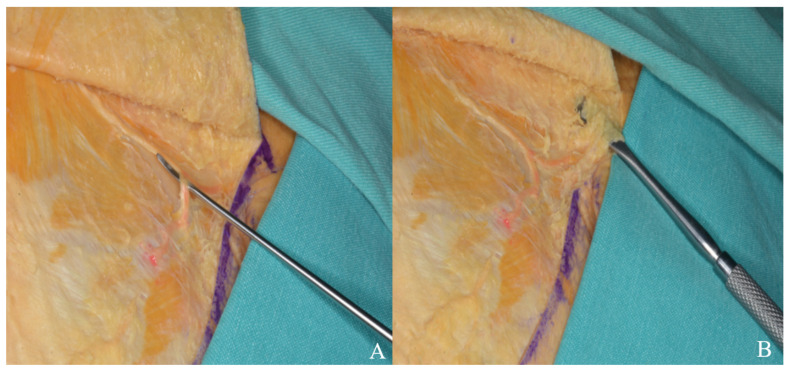
The skin flap was retracted anteriorly, and the superficial temporal artery (**A**) and facial nerve branches (**B**) were carefully dissected and mobilized with a dissector.

To avoid facial nerve injury and preserve the superficial temporal artery, the incision should not extend below the zygomatic arch or more than 1 cm anterior to the tragus [Figure 10].

#### 3.2.2. Temporal Muscle Elevation

**Figure 11:**
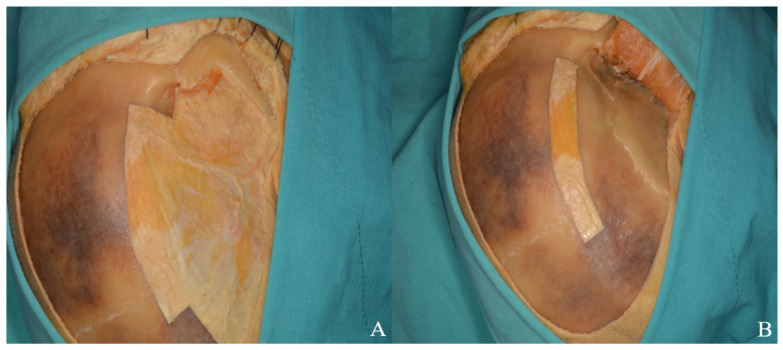
(**A**) The zygomatic arch and temporalis muscle were dissected in a subperiosteal plane, and (**B**) the muscle flap was subsequently repositioned over the bone to achieve anatomical closure of the muscle and fascia.

In the orbitozygomatic approach, the facial nerve is preserved using interfacial or subfascial dissection, like the pterional technique [Figure 3]. The temporal muscle is dissected from the inferior edge of the incision toward the inferior temporal line, leaving a flap at the superior temporal line for reattachment. Subperiosteal dissection then continues inferiorly along the zygomatic arch [Figure 11].

#### 3.2.3. Periorbital Dissection

**Figure 12:**
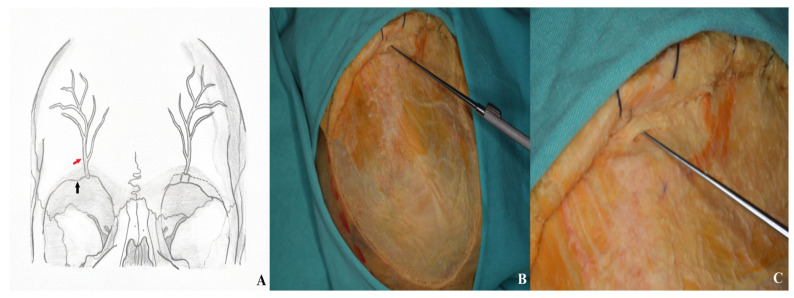
For optimal visualization, the supraorbital nerve was carefully dissected, mobilized laterally, and elevated using a nerve hook [(**A**) supraorbital nerve diagram: black arrow: supraorbital notch; red arrow: supraorbital nerve. (**B**,**C**) Cadaver image].

During temporal muscle dissection, continuity of the periosteum and periorbita along the superior orbital rim must be preserved. The inferior orbital fissure is accessed by carefully elevating the periorbita from the lateral orbital wall, avoiding injury to the lacrimal gland. Dissection proceeds medially to the supraorbital notch, where the supraorbital nerve is identified as it exits the foramen [Figure 12].

#### 3.2.4. Craniotomy

• One Piece Orbitozygomatic Craniotomy

The one-piece approach involves a frontotemporal craniotomy with en bloc removal of the lateral orbital wall, orbital roof, and zygomatic arch.

**Figure 13:**
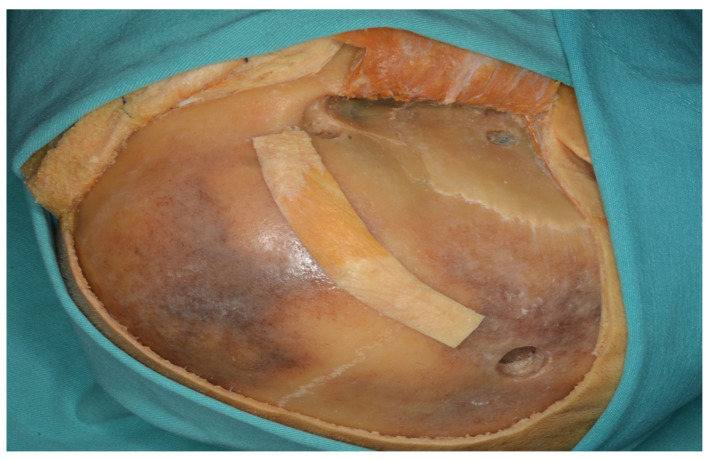
Three burr holes were placed prior to performing a one-piece orbitozygomatic craniotomy.

The first burr hole (McCarty) reveals the frontal dura and periorbita, offering safe access to the anterior cranial fossa and orbit in supraorbital and orbitozygomatic procedures. The second burr hole is placed in the squamous temporal bone above the zygoma root, and the third is drilled in the frontal bone, either lateral to the supraorbital notch or posterior to the superior temporal line [Figure 13].

**Figure 14:**
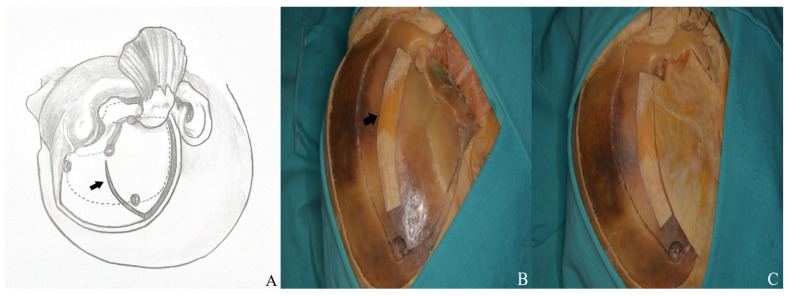
(**A**) Craniotomy design. (**B**) The temporalis muscle was retracted anteriorly to expose the keyhole and allow connection of the anterior and posterior burr holes, and (**C**) the burr holes were then linked using a high-speed drill system [black arrow: temporalis fascia preserved for closure].

**Figure 15:**
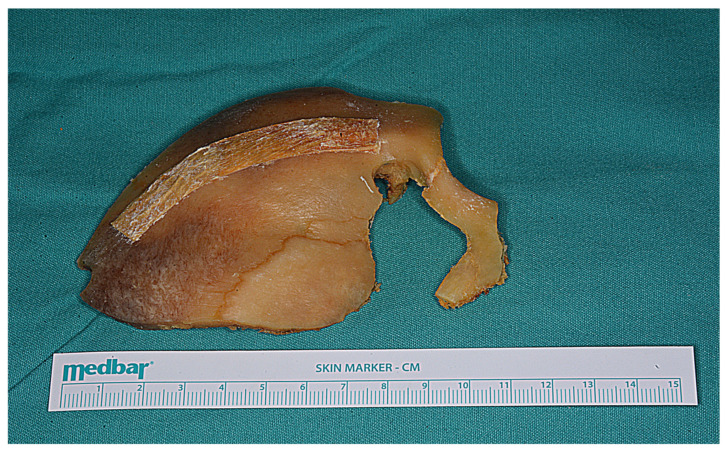
The bone flap was subsequently removed as a one piece.

• In the one-piece orbitozygomatic approach, six osteotomies follow the burr holes [Figure 14 and Figure 15].
• The first extends from the periorbital aspect of the McCarty hole to the inferior orbital fissure.
• The second runs from the zygomatic body to the inferior orbital fissure, ~1 cm below the frontal and temporal zygomatic limbs.
• The third is made at the temporal root of the zygoma, anterior to the articular tubercle.
• The fourth involves the orbital roof, including the supraorbital rim and infraorbital cut; the periorbita must be protected.
• The fifth connects the anterior frontal and posterior temporal burr holes, passing anteriorly while preserving the supraorbital ridge.
• The sixth links the McCarty hole to the posterior temporal burr hole.

• Two Piece Orbitozygomatic Craniotomy

The two-part orbitozygomatic approach combines a supraorbital osteotomy with a pterional craniotomy and partial zygoma removal.

**Figure 16:**
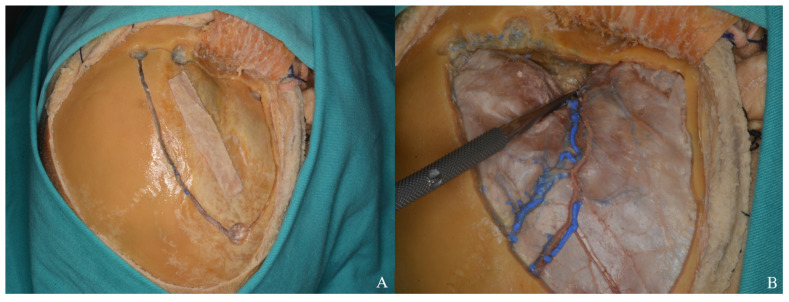
(**A**) Osteotomies were performed using four burr holes in accordance with the standard pterional craniotomy technique. (**B**) The sphenoid ridge was drilled to optimize exposure.

A standard pterional craniotomy is performed using a McCarty hole, a second burr hole in the temporal squama above the zygomatic arch, and a third in the frontal bone or just posterior to the superior temporal line. Rongeurs and a drill are used to access the middle cranial fossa, and the sphenoid wing is drilled laterally to the orbital roof [Figure 16].

**Figure 17:**
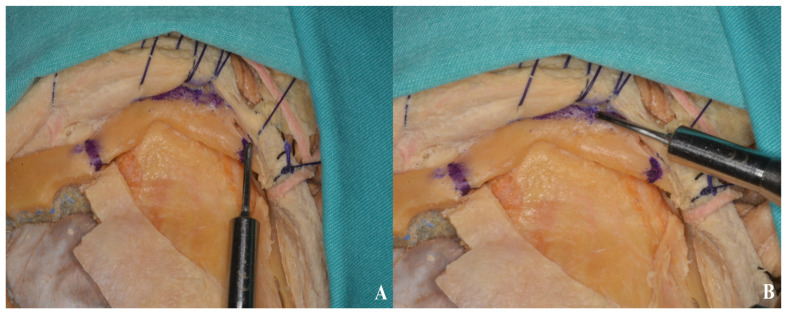
Anterior (**B**) and posterior (**A**) osteotomies of the zygomatic arch.

**Figure 18:**
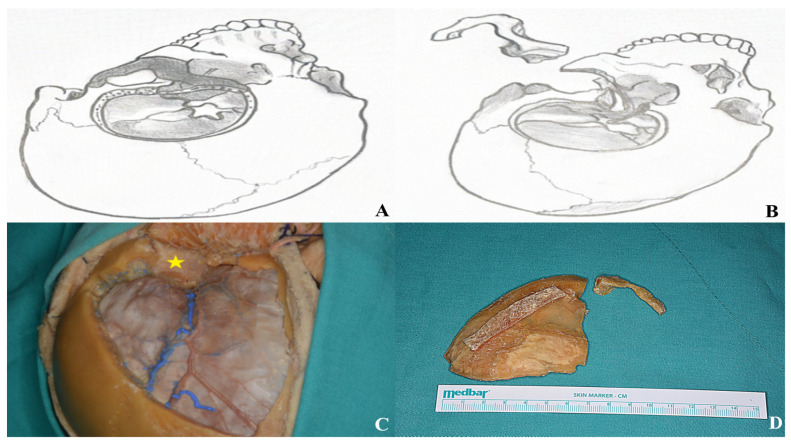
After completing the osteotomies, the bone flaps were carefully removed as part of the two-piece orbitozygomatic approach [(**A**,**B**) two-piece orbitozygomatic cuts; (**C**) cadaver image; yellow star: orbit. (**D**) Bone flaps].

The first osteotomy is made medial to the orbital rim, lateral to the supraorbital ridge, and extends to the medial edge of the craniotomy. The second follows from the posterior end of this cut to the superior orbital fissure. The final orbital cut runs perpendicular to the rim, just lateral to the frontozygomatic suture, toward the superior orbital fissure. Anterior and posterior cuts of the zygomatic arch complete the elevation of the second bone flap [Figure 17 and Figure 18].

#### 3.2.5. Basal Drilling

The sphenoid ridge is a sharp, curved bony landmark on the posterior aspect of the sphenoid lesser wing, extending posteroinferiorly toward the anterior clinoid process. It demarcates the anterior cranial fossa floor from the anterior wall of the middle cranial fossa.

**Figure 19:**
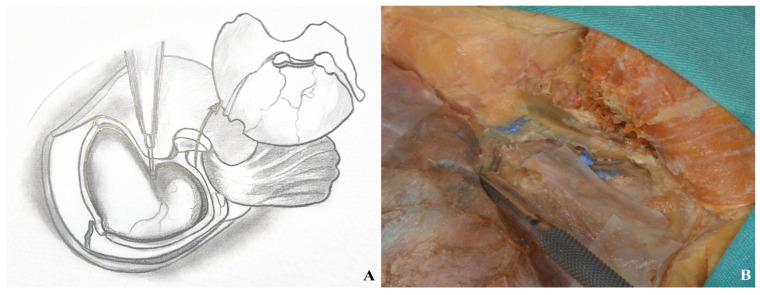
Basal drilling ((**A**) drawing exercises, (**B**) cadaver focus).

To minimize brain retraction and improve skull base exposure, the sphenoid ridge, orbital roof, and squamous temporal bone are resected [Figure 19]. The orbital roof ridges should be removed without violating the periorbita, while sphenoid ridge resection is limited to the meningoorbital artery to prevent anterior clinoid injury.

#### 3.2.6. Dura Incision and Sylvian Fissure Opening

**Figure 20:**
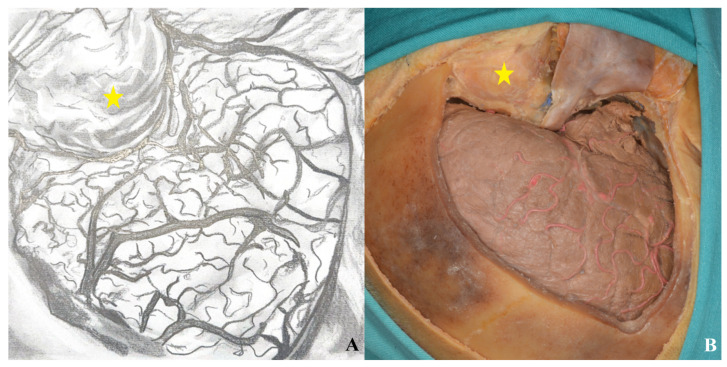
Following the one-piece orbitozygomatic craniotomy, the dura was incised in a curvilinear fashion to expose the underlying cortex ((**A**) drawing exercises, (**B**) cadaver picture, yellow star: orbit).

**Figure 21:**
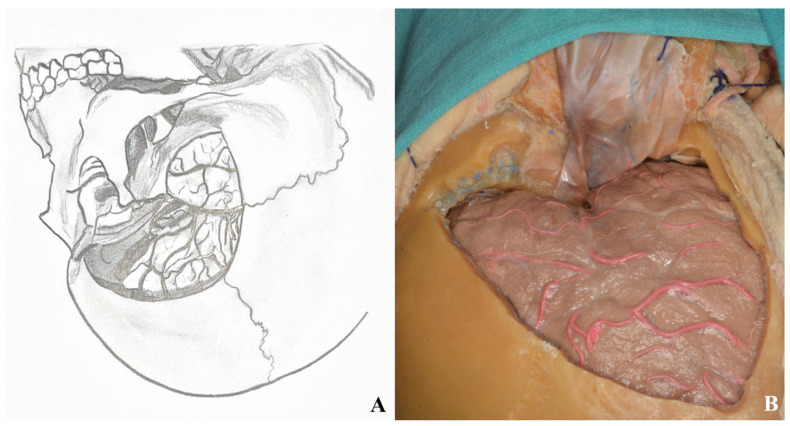
Dura was opened after two-piece orbitozygomatic craniotomy ((**A**) drawing visions, (**B**) cadaver illustration).

A C-shaped dural incision is made extending from the superior orbital rim to the midpoint of the temporal squamous bone and reflected anteriorly. This exposure reveals the inferior and middle frontal gyri, the inferior, middle, and superior temporal gyri, as well as the Sylvian fissure [Figure 20 and Figure 21].

**Figure 22:**
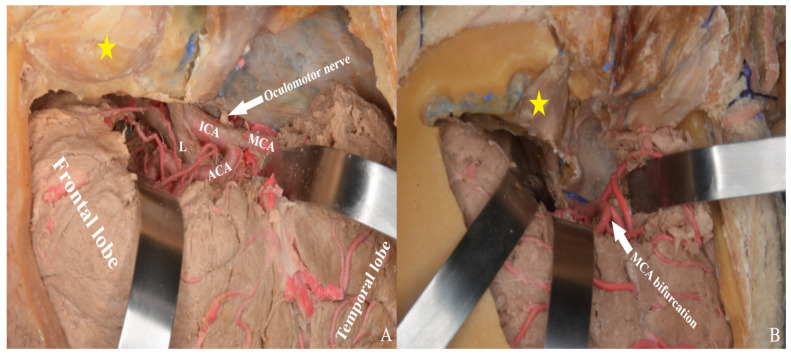
After Sylvian fissure dissection, retraction of the frontal and temporal lobes exposed the optic nerve, vessels, orbit (yellow star), and anterior and middle cranial fossae, allowing access to the lamina terminalis (**A**) one-piece, (**B**) two-piece orbitozygomatic approach (ICA: internal carotid artery; MCA: middle cerebral artery; ACA: anterior cerebral artery; L: lamina terminalis).

Through the transsylvian approach, the Sylvian fissure and anterior basal cisterns are accessed and opened [Figure 22].

**Figure 23:**
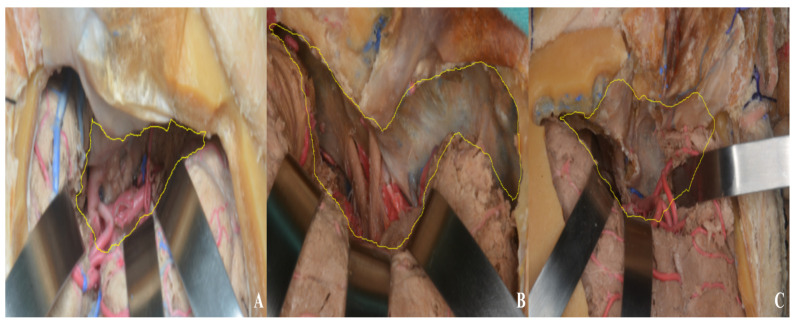
Middle and anterior cranial fossa exposure area ((**A**) pterional approach, (**B**) one-piece orbitozygomatic approach, (**C**) two-piece orbitozygomatic approach; yellow area: exposure).

Finally, the exposure achieved in the anterior and middle cranial fossae was compared across the pterional, one-piece orbitozygomatic, and two-piece orbitozygomatic approaches. Area measurements were performed using ImageJ (1.54g), yielding 9.69 cm^2^ for the pterional approach, 23.92 cm^2^ for the one-piece orbitozygomatic approach, and 28.68 cm^2^ for the two-piece orbitozygomatic approach [Figure 23]. Although a larger number of cadaveric specimens would be required for a statistically robust comparison, the primary aim was to provide neurosurgeons and trainees with a visual illustration of the differences in exposure.

### 3.3. Lamina Terminalis

From superior to inferior, in the coronal plane, the fornix column, foramen of Monroe, anterior commissure, lamina terminalis, and optic chiasm form the anterior wall of the third ventricle. The lamina terminalis forms the anterior wall of the third ventricle and, by adhering broadly and flatly to the optic chiasm, provides the posterior formation of the optic recess [7].

The anterior portion of the lamina terminalis contains the lamina terminalis cistern, which houses the A1 and proximal A2 segments of the anterior cerebral artery, the anterior communicating artery, the Heubner recurrent artery, the hypothalamic arteries, the fronto-orbital arteries, and the anterior cerebral vein [8].

Aneurysmal subarachnoid hemorrhage is a significant cause of death and disability, occurring in 2–32 per 100,000 people in the cerebral hemorrhagic stroke category. Hydrocephalus is a serious cause of mortality and morbidity that can occur in the acute or chronic phase due to aneurysmal subarachnoid hemorrhage. An external ventricular drainage system is used for acute hydrocephalus, while a shunt system is required for chronic hydrocephalus [9].

Fenestration of the lamina terminalis is a useful procedure for reducing intracranial pressure during the clipping of anterior circulation rupture aneurysms. During the operation, the lamina terminalis, which is normally a thin membrane slightly visible posterosuperior to the optic chiasm, is exposed. Fenestration must be performed strictly along the midline, the supply to the optic nerve and chiasm must not be compromised, and the optimal fistula diameter should be between 5 and 6 mm. The most common complication encountered in lamina terminalis fenestration is reclosure of the fistula due to adhesion [9].

In 1995, Yaşargil sharply opened the lamina terminalis to prevent closure due to adhesions and subsequently applied the technique of coagulating the opened edge. Additionally, Yaşargil demonstrated that cisternal blood clearance is important for the functionality of fenestration [10].

In subsequent studies, it has been suggested that the Liliequist membrane should also be opened to ensure that the lamina terminalis fenestration remains more functional; it has been reported that in cases where both the lamina terminalis and Liliequist membrane were opened, the rate of shunt-related hydrocephalus was lower compared to cases where only the lamina terminalis fenestration was performed [11].

### 3.4. Lamina Terminalis Cistern

The lamina terminalis cistern serves as a surgical access guide for anterior communicating artery aneurysms and tumors located posterior to the optic chiasm. The lamina terminalis cistern, described by Yaşargil, contains the anterior communicating artery, Heubner’s recurrent artery, hypothalamic arteries, the proximal portion of the fronto-orbital artery, and the venous structure of the lamina terminalis [12,13].

The upper portion of the lamina terminalis cistern is visible as a space in the anterior-posterior direction. Its lateral walls extend outward inferiorly, and the inferior boundary space is the superior surface of the optic chiasm [13].

The lamina terminalis is located on the posterior and inferoposterior surfaces of the cistern. The posterior gyrus rectus and the septal area in the lamina terminalis cistern form the lateral walls of the cistern [13].

The arachnoid layer surrounding both optic nerves envelops the anterior layer of the lamina terminalis cistern. The pia layer located on the lateral wall of the cistern adheres to each anterior cerebral artery and defines the anterior border of the cistern [13].

The anterior commissure is in the center of the lamina terminalis cistern and adjacent to the optic chiasm, 1 cm above it. Furthermore, in the anterior-posterior direction, the anterior border of the cistern is at the same level as the limbus sphenoidalis [13].

In this study, we present a detailed set of illustrations demonstrating the pterional craniotomy, one-piece, and two-piece orbitozygomatic approaches for accessing the lamina terminalis region.

The pterional craniotomy provides exposure to the anterior and middle cranial base. The accompanying schematic outlines each surgical step and emphasizes key anatomical landmarks, enhancing the understanding of the technique and associated neuroanatomy.

Similarly, the diagrams of the one-piece and two-piece orbitozygomatic approaches depict the sequential steps and relevant anatomical structures, serving as a comprehensive visual reference for these skull base techniques.

## 4. Discussion

We conducted a study to identify the neurovascular structures present in the cistern of the lamina terminalis. This region includes the A1 and A2 segments of the anterior cerebral arteries, the anterior communicating artery, Heubner recurrent artery, subcallosal, and orbitofrontal arteries. We aimed to demonstrate the best approaches for accessing vascular pathologies or tumoral lesions in this region. We utilized three commonly employed approaches: the pterional approach, the single approach, and the two-piece orbitozygomatic approach.

In all three surgical approaches, meticulous preservation of the parietal and temporal branches of the superficial temporal artery is essential. This vessel has gained increased importance in recent years, particularly due to its frequent utilization in contemporary intracranial bypass procedures [14,15,16]. Situated close to the skin surface, the superficial temporal artery can be readily palpated anterior to the tragus. For these approaches, the recommended safe starting point for the incision lies within the deep temporal fascia, between the superior and inferior temporal lines. The safe dissection zone of the deep temporal fascia extends approximately 1–3 cm anterior to the vertical line passing through the tragus [17]. The temporal muscle is enveloped by the superficial fascia, which consists of two distinct layers: a superficial layer and a deep layer. Between these layers lies an interfascial fat pad that contains the branches of the facial nerve [18]. Yaşargil described the interfascial dissection technique to protect the frontotemporal branches of the facial nerve and to minimize postoperative cosmetic deformities [19]. In the subfascial approach, both the superficial and deep layers of the temporal fascia are elevated together from the surface of the temporal muscle, extending from the superior temporal line to the frontal pericranium [20,21].

The pterional approach is a surgical technique that allows access to the cisterna of the lamina terminalis, along with its associated neurovascular structures. Using this approach, it is possible to visualize the carotid bifurcation, basal cistern opening, middle cerebral artery M1 segment with sylvian sphenoidal compartment dissection, and middle cerebral artery M2 segment with opercular-insular dissection [1]. We traced the path of various blood vessels in the brain, including the internal carotid artery bifurcation, anterior cerebral artery A1 segment, anterior communicating artery, middle cerebral artery M1 segment, middle cerebral artery bifurcation, middle cerebral artery M2 segment, posterior communicating artery, and anterior choroidal artery.

The orbitozygomatic approach provides a panoramic view of the anterior skull base with minimal parenchymal retraction. Over the years, there have been several changes made to the literature [3,4,5,22,23,24,25,26]. It has been reported that increasing the width of the surgical area can lead to improved cosmetic outcomes following surgery. This may also result in lower rates of enophthalmos and cerebrospinal fluid fistula [1,23,25,27,28,29]. The 1998 modification by Zabramski involved removing the bone flap in two pieces. This modification allows for a larger surgical area, providing a significant advantage in this approach [25].

Alaywan et al. discovered that an orbitozygomatic craniotomy offers a wider field of view compared to other craniotomy approaches. The field of view is approximately 75% greater than that of a subfrontal craniotomy, 46% greater than that of a pterional craniotomy, and 86% greater than that of a subtemporal craniotomy [27]. In a comparative anatomical cadaver study with the pterional and pretemporal approaches, the orbitozygomatic approach offered significant surgical advantages, particularly in terms of skull base exposure along with the basilar artery. Regarding access to the pathological area, removal of the orbital rim and zygomatic bone provided a quantitatively wider view and was observed to increase surgical freedom in accessing structures with less brain retraction. This anatomical study demonstrated that the orbitozygomatic craniotomy offers significant surgical advantages over the pretemporal approach, particularly when approaching lesions extending to the interpeduncular and prepontine cisterns, the ipsilateral ICA and MCA, and the contralateral sylvian cisterns [30,31,32]. In cases where a wide craniotomy is performed, less brain tissue will need to be removed, leading to better clinical outcomes and improved access to pathology. Nanda et al. demonstrated that the combination of the pterional and anterior temporal approaches resulted in an 8 cm increase in the anteroposterior axis and a 10 cm increase in the superoinferior axis compared to the orbitozygomatic approach [33]. Meybodi and colleagues conducted a study to compare the effectiveness of two microsurgical approaches (orbitozygomatic and subtemporal craniotomy) for treating bacillary crest aneurysms. They concluded that orbitozygomatic craniotomy is a safer method for both patients and surgeons, as it provides better proximal control and dominance of the aneurysm and surrounding neurovascular structures [29]. Yagmurlu et al. demonstrated that mini-orbitozygomatic and mini-pterional approaches in aneurysm surgery result in reduced temporal muscle atrophy and minimized brain parenchymal retraction in appropriately selected patients [6]. Studies have also indicated that the extent of exposure of the anterolateral pons is similar between the pterional and orbitozygomatic approaches. Therefore, the choice between these approaches, particularly for treating pontine cavernomas, should be tailored to individual cases, primarily based on the surgeon’s preference [34].

In a review comparing variants of the orbitomaxillary approach, one-, two-, and three-piece craniotomies were examined. Superolateral orbitotomy widens the subfrontal and transsylvian corridors and increases surgical freedom toward the basal forebrain, hypothalamic region, interpeduncular fossa, and basilar apex. Zygomatic osteotomy shortens the working distance to the main targets of the pretemporal and subtemporal routes. Removal of the orbitomaxillary bar eliminates the need for brain retraction and allows for multi-angle trajectories. Orbitozygomatic approaches can be customized according to the location and extent of the lesion, thereby optimizing target exposure and reducing the potential for complications [35].

The frontotemporal and orbitozygomatic routes grant excellent access to anterior and middle skull base abnormalities as well as the infratemporal fossa. Crucial considerations include proper positioning of the head, planning the skin incision, retracting the scalp, fat pad dissection, and facial nerve preservation. Further essential steps are the creation of either a true or false McCarty keyhole, drilling of the sphenoid wing, anterior clinoidectomy, finishing the craniotomy along with auxiliary orbital osteotomy cuts, dural opening, and intradural access to neurovascular structures [36].

Additionally, the use of cadaver-free models, such as the UpSurgeOn training application, in conjunction with the orbitozygomatic and pterional approaches, provides a comprehensive approach to neurosurgery education through practical simulations [37].

As part of our study, we obtained images of single-piece, two-piece orbitozygomatic, and pterional craniotomy techniques used to access the lamina terminalis cistern. Our aim was to conduct a study focusing on the anatomical orientation of pterional, single-piece, and two-piece orbitozygomatic craniotomies to reach the lamina terminalis cistern.

As a limitation, our study was performed on formalin-fixed human cadavers and silicone-injected vascular structures. The neuroanatomical structures of fixed specimens are very suitable for dissection. The structures are easily distinguishable and correspond well to their actual anatomical locations. However, parenchymal excision is considerably more difficult than in fresh cadavers and living tissue. Therefore, the surgical field will be easier to visualize in actual surgery.

Additionally, this research focuses on a microsurgical approach. The primary aim was to provide detailed, step-by-step demonstrations, accompanied by photographic documentation, of the pterional and orbitozygomatic approaches to the lamina terminalis cistern, using both techniques, to assist neurosurgeons and neurosurgery residents in accessing the lamina terminalis cistern. Therefore, it was not deemed necessary to include a large number of cases. However, variables such as neurovascular anatomical variations could be further investigated in future studies using a much larger sample cohort.

## 5. Conclusions

The pterional and orbitozygomatic craniotomies are among the most frequently performed approaches to the lamina terminalis region and constitute the foundational techniques taught in neurosurgical training programs. Mastering the sequential steps from skin incision to skull base exposure, as well as their clinical applications, is essential for neurosurgeons and residents at various stages of training. Given the adaptability of the pterional approach and the one- and two-piece orbitozygomatic techniques, microsurgical laboratory practice plays a vital role during the learning process. Therefore, we believe that our study will serve as a valuable reference for neurosurgeons in accessing the lamina terminalis cistern using all three approaches.

## Data Availability

The original contributions presented in this study are included in the article. Further inquiries can be directed to the corresponding author.
